# Determinants of short birth interval among married multiparous women in Chinaksen district, eastern Ethiopia: a case-control study

**DOI:** 10.3389/fgwh.2023.1278777

**Published:** 2024-01-08

**Authors:** Bekry Aleye, Ahmedin Aliyi Usso, Bezatu Mengistie, Yadeta Dessie, Hassen Abdi Adem, Addisu Alemu, Mohammed Yuya, Aminu Mohammed

**Affiliations:** ^1^ East Hararghe Health Office, Oromia Health Bureau, Addis Ababa, Ethiopia; ^2^ School of Nursing and Midwifery, College of Health and Medical Sciences, Haramaya University, Harar, Ethiopia; ^3^ School of Public Health, Saint Paul Hospital Millennium Medical College, Addis Ababa, Ethiopia; ^4^ School of Public Health, College of Health and Medical Sciences, Haramaya University, Harar, Ethiopia; ^5^ Department of Midwifery, College of Health and Medical Sciences, Dire Dawa University, Dire Dawa, Ethiopia

**Keywords:** optimal birth interval, determinants, married women, semi-pastoral community, Ethiopia

## Abstract

**Background:**

The short birth interval is a common public health issue that affects women's and children's health in sub-Saharan Africa. Despite a higher burden of short birth intervals reported in Ethiopia, there is limited evidence to indicate the primary risk factors, particularly in rural eastern Ethiopia. Therefore, this study assessed the determinants of the short birth interval among married multiparous women in Chinaksen district, Eastern Ethiopia.

**Methods:**

A community-based case-control study was conducted among randomly selected 210 cases and 210 controls from April 01 to June 30, 2019. The total sample size (219 cases and 219 controls) were calculated using Epi-Info software version 7.2. Data were entered using EpiData version 3.1 and analyzed using SPSS version 27, and multivariable logistic regression analyses conducted to identify the determinants of short birth intervals. Adjusted odds ratio (AOR) with a 95% confidence interval (CI) was used to report the strength of association and statistical significance declared at *p-*value < 0.05.

**Results:**

The women in the young age group (AOR = 2.33, 95% CI: 1.03, 5.26), missed their antenatal care visits (AOR = 2.23, 95% CI: 1.18, 4.21), failed to utilize postpartum contraceptives (AOR = 5.98, 95% CI: 3.62, 9.89), did not attend postnatal care visit (AOR = 1.86, 95% CI: 1.13, 3.05), nonexclusive breastfed (AOR = 4.05, 95% CI: 2.18, 7.52), short and medium period of breastfeeding (AOR = 4.00, 95% CI: 1.34, 12.10) and (AOR = 3.56, 95% CI: 1.62, 7.82), respectively and female sex of preceding child (AOR = 1.92, 95% CI: 1.18, 3.12) were the important risk factors of short birth interval.

**Conclusions:**

Women's age, antenatal care visits, postnatal care attendance, utilization of postpartum contraceptives, exclusive breastfeeding practice, duration of breastfeeding, and sex of the preceding child were the primary predictors of short birth intervals. Improving the utilization of maternal healthcare services in health facilities would be imperative to prevent and reduce short birth intervals, and its negative consequences.

## Introduction

The period between two successive live births is known as the birth interval. The World Health Organization (WHO) states that the ideal birth interval is between two consecutive live births of 33–59 months, whereas a short birth interval is between two successive live births of less than 33 months (1). Short birth interval is a severe public health issue that affecting about 25% of live births worldwide. The problem is most common in developing countries that 33% of short birth intervals occurring in Central Asia and 57% in Sub-Saharan Africa (SSA) (2). Ethiopia is another country where the issue is more serious, affecting 53% of live births each year (3).

The short birth interval is the most important factor influencing maternal, neonatal, and child health outcomes (4). Short birth intervals also increase the likelihood of unfavorable pregnancy outcomes such as premature birth, low birth weight, intrauterine growth restriction (5–7), obstructed labor, maternal hemorrhage, anemia, and cesarean birth (1, 4). Globally, an estimated 20.5 million neonates are born with low birth weight each year, and of these, 48% (9.84 million) from Southeast Asia, 24% (4.92 million) from sub-Saharan Africa, and 17% (3.49 million) from Ethiopia (8, 9). In addition, short birth intervals raise the risks of maternal, neonatal, and child morbidity, mortality, and disability (5, 10, 11). It increases the risk of neonatal death by 2.3, perinatal mortality by 3.8, and maternal mortality by 1.7 times. Accordingly, the neonatal mortality rate per 1,000 live births was 18 in the world, 27 in SSA, and 92 in Ethiopia (3, 12). The maternal mortality rate per 100,000 live births was 210 in the world, 231 in Lower and Middle Income countries (LMIC), 534 in SSA, and 412 in Ethiopia (13, 14). On the other hand, short birth interval has a major contribution to increasing the overall fertility rate in developing countries. The total fertility rate was 2.31 births per woman in the world (15), and the rate is much higher in SSA (4.7) (16) and Ethiopia (4.6) (14).

The Ethiopia Federal Ministry of Health suggested the optimal birth interval of 33–59 months between two consecutive pregnancies recommended for promoting women's and children's health (17). However, around 53% of multiparous women in Ethiopia had short periods between consecutive live births (14). A previous study indicated that 56% of women had short birth intervals in rural eastern Ethiopia (18).

Previous studies suggested that women demographic characteristics, including their age at first marriage, occupation, educational status, and wealth index, were predictors of short birth intervals (19, 20).

The previous risk factor studies were analyzed secondary data (21) and addressed the significance of short birth intervals (18), but there is a scarcity of information on their major preventable determinants. Additionally, some previous studies used a birth interval of less than 36 months as the cutoff point and parameter to diagnose short birth intervals (20, 22), and they used a cross-sectional design (23), which is unsuitable for identifying factors that contribute to short birth intervals. Despite the higher reported prevalence of short birth intervals, evidence showed that the main risk factors for short birth intervals were scarce in rural eastern Ethiopia. Given that, it is essential to comprehend the underlying causes that are affecting the short birth interval in a different manner. Therefore, this study revealed determinants of short birth intervals among married multiparous women in semi-pastoral communities of Chinaksen District, Eastern Ethiopia.

## Methods and materials

### Study design and setting

A community-based case-control study was conducted from April 01 to June 30, 2019, in Chinaksen district. Chinaksen is one of 20 districts in the East Hararghe Zone, located 659 kilometers (km) east of Addis Ababa, the capital city of Ethiopia, and a semi-pastoral area in rural eastern Ethiopia. Administratively, the district was divided into three urban and 49 rural kebeles, with 119,123 estimated total population in 2018, 26,217 women of reproductive age group, and 4,284 pregnant women. According to the district health office annual report 2018, there are 49 health posts and eight health centers offering general healthcare services.

### Population and sampling

The source population was all married women in the Chinaksen district who had at least two consecutive live births within the five years preceding data collection. The married multiparous women in randomly selected kebeles who had at least two successive live births in the previous five years and who were permanent residents of the district were included in the study. Critically ill and mentally ill women who were unable to respond to interviews, those who had twin births, who had caesarian delivery, who had a history of preceding neonatal death or abortion, and who did not live with their husbands within the last five years were excluded from the study. Cases were married women with birth intervals of less than 33 months who had at least two consecutive live births within the previous five years in randomly selected kebeles of the Chinaksen district, whereas controls were those who had a birth interval of 33 months of two subsequent live births during the data collection period.

Epi-Info software version 7.2 was used to calculate the total sample size (*n* = 438) using a two-population proportion formula (unmatched case-control study) with the following assumptions: a 95% confidence level, 80% power, 5% margin of error, one-to-one controls to cases ratio, 69% proportion of exposed control, AOR of 1.99 (19), and 10% non-response proportion. The final sample size needed for this study was 438 (Cases 219 and Controls 219).

**Figure 1:**
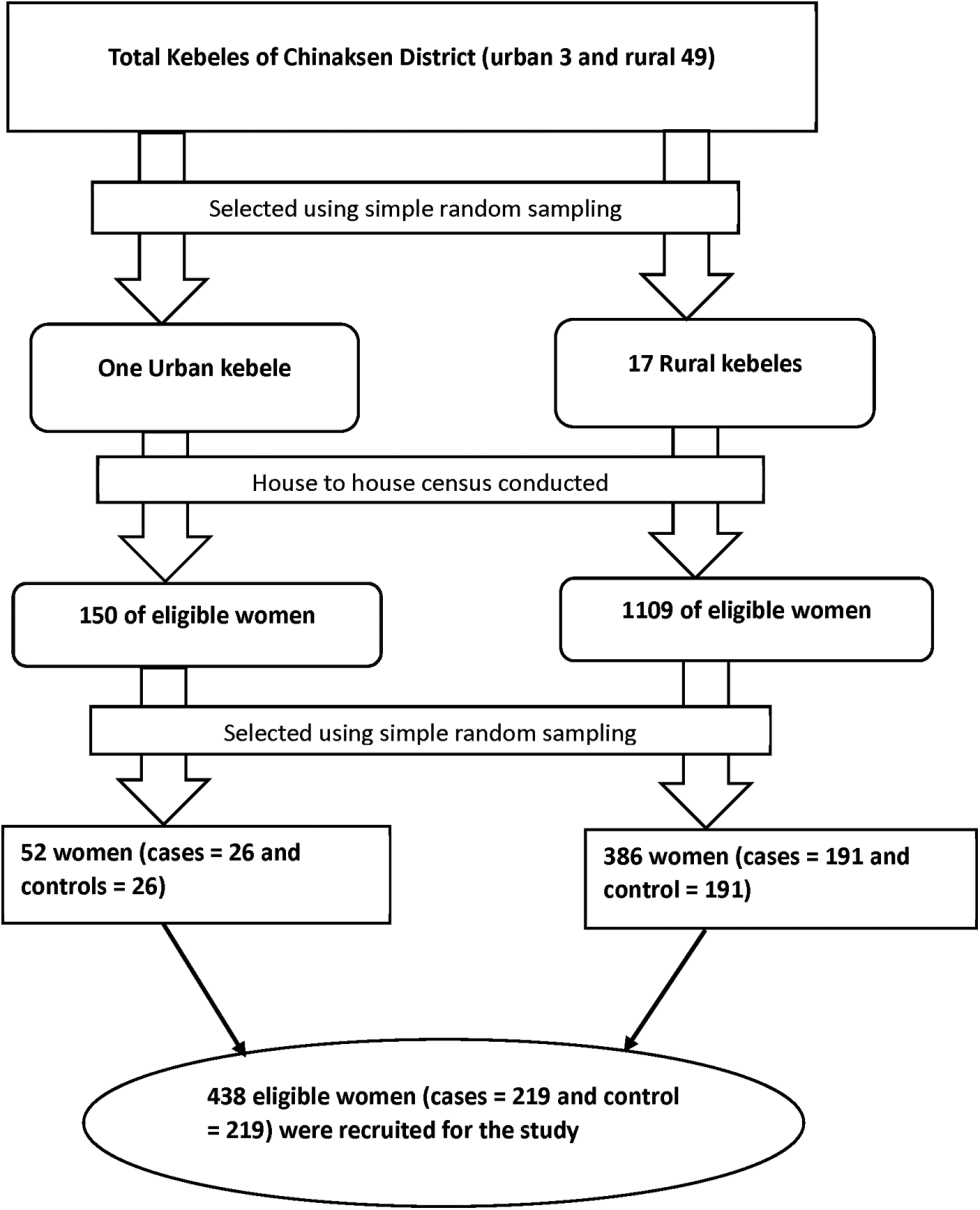
A flow diagram of sampling procedure.

A multistage stratified sampling technique was used to identify the study participants. *First*, we divided kebeles into urban (*n* = 3) and rural (*n* = 49) kebeles. *Second,* one urban and 17 rural kebeles were randomly selected. The house-to-house census conducted in randomly selected kebeles, and eligible households (cases and controls) were identified by recording the birth dates of the last two children. Birth certificates and immunization cards were used to determine the children's birth dates, and health extension workers were consulted for those without birth certificates and immunization cards. Households with eligible women were assigned identification numbers to construct a sampling frame, and a total of 1,259 eligible households (682 cases and 577 controls) identified. *Then*, separate sampling frames build for the cases and controls in each kebeles. *Finally*, the estimated sample size was distributed proportionally to each kebeles (based on the actual number of cases and controls), and eligible participants were recruited using a simple random sampling technique. Participants not present for at least three data collection trips were considered non-respondents. When there were two or more eligible women in one selected household, only one eligible woman was selected using the lottery method (Figure 1).

### Data collection tool and measurement

Data were collected from both cases and controls using pretested structured questionnaires adapted from relevant published literatures (3, 19, 20, 24–26). The questionnaire includes socio-demographic information, reproductive health factors, healthcare-related factors, and birth interval (case/control). The questionnaire first developed in English was translated into the local language (Afan Oromo), and returned back to English by two professionals fluent in both languages. Eighteen trained data collectors collected the data through house-to-house visits using face-to-face interviews. Six trained supervisors with principal investigator were supervised the overall data collection process.

#### Decision-making autonomy

It was measured by four dichotomous (yes/no) items (Cronbach ɑ = 0.72) asking about women's household decision-making autonomy, and each item coded “1” when answered “yes” and coded “0” when answered “no”. The women had low decision-making autonomy when scored “0,” medium decision-making autonomy when scored “1–3,” and high decision-making autonomy when scored “4” (27–29).

#### Wealth index

It was calculated using a standard instrument with 36 dichotomous (yes/no) items asking about domains of the family's wealth level (domestic animals, durable assets, other productive assets, and housing circumstances) (14). We found strong internal consistency of items (Cronbach ɑ = 0.81) and principal component analysis utilizing the varimax rotation approach to estimate composite wealth index factors and the wealth status of the cases and controls.

### Data quality control

Standard questionnaires adapted from validated scales and relevant published literature were used to maintain the data quality. The questionnaire first prepared in English was translated into the local language (Afan Oromo) by two experts who certified in both languages and back to English. We pretested the questionnaires on 5% of the total sample size (11 cases and 11 controls) to ensure validity in a non-selected kebele (Kalaroga) in the district, and the revisions were made, accordingly. Eighteen data collectors were trained to collect the data under supervision six supervisors after receiving training for one days on the objective of the study and the data collection technique. Before beginning the statistical analysis, composite index scores were generated and tested, which could improve the validity of the measurements and associated computed indices and the estimates were used in the study.

### Data processing and analysis

After ensuring completeness and consistency, the data was entered into EpiData version 3.1 for control data entry and then exported to SPSS version 27 for analysis. Descriptive statistics such as frequency, measure of central tendency, and measure of dispersions were employed to characterize the case and control populations. Before analysis, the internal consistency of the items was checked for composite index scores for decision-making autonomy (Cronbach ɑ = 0.72) and wealth index (Cronbach ɑ = 0.81) using reliability analysis.

Bivariable logistic regression analyses were conducted to determine the factors associated with short birth intervals among married women. Multivariable logistic regression analyses were fitted to determine the significant risk factors for short birth intervals using a backward stepwise (Likelihood Ratio) method of model building, and the overall model adequacy was confirmed using the Hosmer and Lemeshow goodness of fit test (*p*-value >0.05). Adjusted Odds Ratio (AOR) with a 95% confidence interval (CI) was used to report the strength of the association, and the statistical significance declared at a *p*-value <0.05.

## Results

### Characteristics of participants

A total of 210 cases and 210 controls with a 96% response rate participated in the study. The median age and interquartile range (IQR) of cases and controls were 28 years (IQR = 8; 25th percentile = 27 and 75th percentile = 35) and 30 years (IQR = 6; 25th percentile = 24 and 75th percentile 30), respectively, with 59.0% of cases and 60.0% of controls were between the ages of 25 and 34 years old. The majority (74.3%) of cases and 73.3% of controls had no formal education, and about 86.2% of cases and 78.1% of controls were housewives in their occupation. Almost all (97.1%) of cases and 95.7% of the controls were Muslim by religion (Table 1).

**Table 1:** Socio-demographic characteristics of participants in Chinaksen district, eastern Ethiopia, 2019 (*n* = 420).

Characteristics	Categories	Cases (%)	Controls (%)
Age (in years)	≤24	54 (25.7)	26 (12.4)
	25–34	124 (59.0)	126 (60.0)
	≥ 35	32 (15.2)	58 (27.6)
Residence area	Rural	189 (90.0)	184 (87.6)
	Urban	21 (10.0)	26 (12.4)
Ethnicity	Oromo	176 (83.8)	162 (77.1)
	Somale	22 (10.5)	31 (14.8)
	Amhara	9 (4.3)	14 (6.7)
	Gurage	3 (1.4)	3 (1.4)
Religion	Muslim	204 (97.1)	197 (93.8)
	Orthodox	6 (2.9)	10 (4.8)
	Protestant	0 (0.0)	3 (1.4)
Education	No formal education	156 (74.3)	144 (68.6)
	Primary school	43 (20.5)	40 (19.0)
	Secondary school	5 (2.4)	8 (3.8)
	College and above	6 (2.9)	8 (8.6)
Husband education	No formal education	140 (66.7)	119 (56.7)
	Primary school	54 (33.3)	73 (43.3)
	Secondary school	6 (2.9)	4 (1.9)
	College and above	10 (4.8)	14 (6.7)
Occupation	Housewife	181 (86.2)	164 (78.1)
	Merchant	19 (9.0)	32 (15.2)
	Employed	10 (4.8)	14 (6.7)
Husband occupation	Farmer	164 (78.1)	165 (78.6)
	Merchant	27 (12.9)	16 (7.6)
	Employee	14 (6.7)	22 (10.5)
	Daily labor	5 (2.4)	7 (3.3)
Wealth index	Poor	67 (37.9)	75 (35.7)
	Medium	83 (39.5)	76 (36.2)
	Rich	60 (28.6)	59 (28.1)
Decision making autonomy	Low	68 (32.4)	33 (15.7)
	Medium	93 (44.3)	123 (58.6)
	High	49 (23.3)	54 (25.7)

Sixty-one (29.0%) of cases and fifty-four (26.7%) of controls were married before the age of 18 years old, with the median age at first marriage and IQR for both cases and controls were 19 (IQR = 3; 25th percentile = 17 and 75th percentile = 20) years old. About 47.6% of cases and 39.5% of the controls were given the first childbirth at less than 20 years old, with the median age at first childbirth and IQR for both cases and controls being 18 years old(IQR = 3; 25th percentile = 18 and 75th percentile = 21). The majority (69.5%) of cases and 90% of controls received antenatal care visits during the previous pregnancy. Around 79.0% of cases and 29.5% of the controls did not utilize postpartum contraceptives during the preceding birth. Among delivery places of a preceding childbirth, 61.0% of cases and 29.0% of controls were home delivery, and about 59.5% of cases and 30.0% of controls did not attend postnatal care visits during the preceding birth. The majority (60.0%) of cases and 89.5% of controls were exclusively breastfed the preceding child, and around 6.7% of cases and 21.0% of controls breastfed for greater than 24 months. Regarding the sex of the previous child, 56.7% of cases and 37.1% of controls were female. Nearly one in every four (23.3%) cases and one in every four (25.7%) controls had higher decision-making autonomy (Table 2).

**Table 2:** Reproductive and health care characteristics of the study participants in chinaksen district, eastern Ethiopia, 2019 (*n* = 420).

Reproductive characteristics	Categories	Cases (%)	Controls (%)
Age at first marriage	≥ 18	149 (71.0)	154 (73.3)
	<18	61 (29.0)	56 (26.7)
Age at first delivery	<20 years	100 (47.6)	83 (39.5)
	≥ 20 years	110 (52.4)	127 (60.5)
Parity	≤ 4	152 (72.4)	136 (64.8)
	> 4	58 (27.6)	74 (35.2)
Number of live children	≤2	93 (44.3)	58 (27.6)
	3–4	70 (33.3)	86 (41.0)
	≥5	47 (22.4)	66 (31.4)
ANC attendance of previous pregnancy	No	64 (30.5)	21 (10.0)
	Yes	146 (69.5)	189 (90.0)
Place of previous delivery	Home	128 (61.0)	61 (29.0)
	Health facility	82 (39.0)	149 (71.0)
PNC visit of preceding birth	No	125 (59.5)	63 (30.0)
	Yes	80 (40.5)	147 (70.0)
PPC use following preceding birth	No	166 (79.0)	62 (29.5)
	Yes	44 (21.0)	148 (70.5)
EBF of preceding child	No	84 (40.0)	22 (10.5)
	Yes	126 (60.0)	188 (89.5)
Breastfeeding duration of preceding child (in months)	≤12	30 (14.3)	11 (5.2)
	13–24	166 (79.0)	155 (73.8)
	>24	14 (6.7)	44 (21.0)
Sex of preceding child	Male	91 (43.3)	132 (62.9)
	Female	119 (56.7)	78 (37.1)
Intention of last child	Unintended	71 (33.8)	21 (10.0)
	Intended	139 (66.2)	189 (90.0)
HDA meeting attendance	No	115 (54.8)	69 (32.9)
	Yes	95 (45.2)	141 (67.1)

ANC, antenatal care; EBF, exclusive breastfeeding; HDA, health development army; PNC, postnatal care; PPC, postpartum contraceptives.

### Determinants of short birth interval

Women’s age, Antenatal care (ANC) attendance, Postnatal Care (PNC) attendance, Postpartum contraceptives (PPC) use, Exclusive Breastfeeding (EBF), Duration of breastfeeding, and sex of preceding child were the determinants of short birth intervals.

Women in the less than 24 years age group were two times more likely (AOR = 2.33, 95% CI: 1.03, 5.26) to have short birth intervals than those in the greater than 34 years age group. Women who did not receive ANC visits during the preceding pregnancy were two times (AOR = 2.23, 95% CI: 1.18, 4.21) more likely to have short birth intervals compared to their counterparts. Women who did not utilize PPC during their previous childbirth were almost six times (AOR = 5.98, 95% CI: 3.62, 9.89) more likely to have short birth intervals than those who utilized PPC. The odds of a short birth interval were 1.86 times higher among women who did not attend PNC visits during the previous birth (AOR = 1.86, 95% CI: 1.13, 3.05) compared to those who attended PNC visits. The odds of a short birth interval were four times higher among women who did not practice exclusive breastfeeding their previous child (AOR = 4.05, 95% CI: 2.18, 7.52) than those who had exclusively breastfed their previous child. Breastfeeding duration of the preceding child of less than or equal to 12 months and 13–24 months increased the risks of the short birth interval by 4 and 3.5 times, respectively (AOR = 4.00, 95% CI: 1.34, 12.10) and (AOR = 3.56, 95% CI: 1.62, 7.82), compared to breastfeeding duration of greater than 24 months. The odds of short birth intervals among women who have been female preceding a child were 1.9 times (AOR = 1.92, 95% CI: 1.18, 3.12) higher than those who had a male preceding child (Table 3).

**Table 3:** Determinants of short birth intervals among married multiparous women in Chinaksen district, eastern Ethiopia, 2019 (*n* = 420).

Characteristics	Category	Cases *n* (%)	Controls *n* (%)	COR (95% CI)	AOR (95% CI)
Age (in years)	≤24	54 (25.7)	26 (12.4)	3.76 (1.99, 7.11)***	2.33 (1.03, 5.26)*
	25–34	124 (59.0)	126 (60.0)	1.78 (1.08, 2.93)*	1.29 (0.69, 2.41)
	≥ 35	32 (15.2)	58 (27.6)	1	1
Educational status	No formal education	156 (74.3)	144 (68.6)	3.25 (1.25, 8.41)*	1.71 (0.52, 5.56)
	Primary school	43 (20.5)	40 (19.0)	3.22 (1.16, 8.94)*	2.15 (0.61, 7.62)
	Secondary school	5 (2.4)	8 (3.8)	1.87 (0.44, 7.99)	4.02 (0.76, 21.30)
	College and above	6 (2.9)	18 (8.6)	1	1
Wealth index	Poor	67 (31.9)	75 (35.7)	0.88 (0.54, 1.43)	0.52 (0.27, 1.01)
	Medium	83 (39.5)	76 (36.2)	1.07 (0.67, 1.73)	0.69 (0.37, 1.28)
	Rich	60 (28.6)	59 (28.1)	1	1
Decision making autonomy	Low	68 (32.4)	33 (15.7)	2.27 (1.29, 4.01)***	1.70 (0.82, 3.50)
	Medium	93 (44.3)	123 (58.6)	0.83 (0.52, 1.33)	0.92 (0.51,1.67)
	High	49 (23.3)	54 (25.7)	1	1
ANC attendance of previous pregnancy	No	64 (30.5)	21 (10.0)	3.94 (2.30, 6.76)***	2.23 (1.18, 4.21)*
	Yes	146 (69.5)	189 (90.0)	1	1
Parity	≤ 4	152 (72.4)	136 (64.8)	1.43 (0.94, 2.16)	1.07 (0.53, 2.53)
	> 4	58 (27.6)	74 (35.2)	1	1
Delivery place of previous birth	Home	128 (61.0)	61 (29.0)	3.81 (2.54, 5.73)***	1.56 (0.92, 2.64)
	Facility	82 (39.0)	149 (71.0)	1	1
PPC following previous birth	No	166 (79.0)	62 (29.5)	9.01 (5.77,14.06)***	5.98 (3.62, 9.89)***
	Yes	44 (21.0)	148 (70.5)	1	1
PNC visit of preceding child	Yes	125 (59.5)	63 (30.0)	3.43 (2.29, 5.14)***	1.86 (1.13, 3.05)*
	No	85 (40.5)	147 (70.0)	1	1
EBF of preceding child	No	84 (40.0)	22 (10.5)	5.70 (3.38, 9.59)***	4.05 (2.18, 7.52)***
	Yes	126 (60.0)	188 (89.5)	1	1
Breast feeding duration (in months)	≤12	30 (14.3)	11 (5.2)	8.57 (3.43, 21.42)***	4.00 (1.34, 12.10)*
	13–24	166 (79.0)	155 (73.8)	3.37 (1.77, 6.38)***	3.56 (1.62, 7.82)**
	>24	14 (6.7)	44 (21.0)	1	1
Sex of preceding child	Female	119 (56.7)	78 (37.1)	2.21 (1.50, 3.27)***	1.92 (1.18, 3.12)**
	Male	91 (43.3)	132 (62.9)	1	1
Participate on HDA meeting	No	115 (54.8)	69 (32.9)	2.47 (2.66, 3.67)***	1.29 (0.75, 2.23)
	Yes	95 (45.2)	141 (67.1)	1	1

ANC, antenatal care; AOR, adjusted odds ratio; COR, crud odds ratio; EBF, exclusive breastfeeding; HDA, health development army; PNC, postnatal care; PPC, postpartum contraceptives.

*p < 0.05; **p < 0.01; ***p < 0.001.

Bold values indicate for significant variables.

## Discussion

Although a higher burden of short birth intervals was reported in SSA countries, including Ethiopia, the evidence determining the main risk factors of short birth intervals was insufficient in rural eastern Ethiopia. Therefore, this study identified factors associated with short birth intervals among multiparous women in the Chinaksen district in Eastern Ethiopia. The women's age, ANC attendance, PNC attendance, PPC use, EBF practice, breastfeeding duration, and sex of the preceding child were the determinants of short birth intervals.

This study indicated that the odds of a short birth interval among women who were less than or equal to 24 years old were two times higher than those who were greater than 34 years old. This finding was supported by findings of studies conducted in Mieso, eastern Ethiopia (19), Dembecha, northwest Ethiopia (30), Dessie, northern Ethiopia (31), and Bangladesh (5). Early marriage is more common in rural eastern Ethiopia due to cultural norms. Younger women are more sexually active and fertile; however, they are less likely to use reproductive health services, including contraceptives, for birth spaces in semi-pastoral communities. In addition, young women could not decide to use modern contraceptives without their husband's permission. It is worrisome that young women were more likely to be affected by short birth intervals. Encouraging and empowering young women to utilize reproductive health services is essential for reducing and preventing short birth intervals (32).

The finding of this study revealed that ANC attendance was significantly associated with short birth intervals. Women who did not receive ANC during the preceding pregnancy were two times more likely to have short birth intervals as compared to those who received ANC. This could be a fact because the women who utilized reproductive health services from health facilities might counseled on optimal birth interval and postpartum contraceptive utilization.

The use of postpartum contraceptives protects against short birth intervals. Given that the women missing an opportunity to use postpartum contraceptives during the previous birth were five times more likely to have short birth intervals compared to their counterparts. This finding was consistent with studies done in southern Ethiopia (26), northwest Ethiopia (33), northern Ethiopia (34), eastern Sudan (25), and Nigeria (35). This could be because the use of postpartum contraception can reduce and prevent the chances of unwanted pregnancy, lowering fertility and lengthening the birth interval (36). The mechanisms should established in place to increase women's intentions to utilize immediate postpartum contraception (32).

This study also revealed postnatal care visit was a significant predictor of short birth interval. The odds of having a short birth interval among women who did not receive postnatal care were higher compared to their counterparts. This outcome was in agreement with that of a study conducted in southern Ethiopia (37). Women who did not receive postnatal care may have missed the opportunity to use maternal health care services and receive health information on unwanted pregnancy and preventive techniques.

In addition, this study found that women who did not exclusively breastfeed their preceding child were four times more likely to have short birth intervals than their counterparts; this could be because exclusive breastfeeding causes lactation amenorrhea, which decreases the likelihood of pregnancy owing to hormonal influences. The duration of breastfeeding was also a major risk factor for short birth intervals. Women who breastfed for a short period had a higher risk of having a short birth interval than those who breastfed for more than 24 months. This finding was in agreement with the study conducted in different regions of Ethiopia (19, 23, 30, 31, 38), Kenya (39), Nigeria (35), and Myanmar (24). This could be because breastfeeding has a contraceptive effect due to negative feedback processes in the hypothalamic-pituitary-ovarian axis.

Furthermore, women with the female sex of the preceding child were more likely to have short birth intervals than those with the male sex of the preceding child. The finding was consistent with similar studies conducted in Ethiopia (19, 20, 31). Women who had female children from previous births were eager to become pregnant again till they got a son child.

The study's strength is that it used a community-based case-control study design to evaluate the predictors of short birth intervals in a semi-pastoral community. The study's limitation is because the women respond to some questions concerning earlier birth events, recall bias might affect a trues relationship between exposure and outcome. The study did not consider qualitative data, and hence, this study was conducted among semi-pastoral communities; the results may not be representative of married multiparous women in Ethiopia.

## Conclusions

This study indicated that young age, failure to attend ANC visits, missed opportunity to use postpartum contraceptives, did not receive postnatal care services, nonexclusive breastfeeding, a short duration of breastfeeding, and female sex of the preceding child were independent risk factors of short birth interval. Encouraging all women in the reproductive age group to utilize respectful maternal healthcare services during the antepartum, intrapartum, and postpartum periods would be essential for preventing and reducing the burden of short birth intervals, and its negative consequences. In addition, encouraging optimal breastfeeding practice at the community level would be needed to reduce and prevent short birth intervals.

## Data Availability

The raw data supporting the conclusions of this article will be made available by the authors, without undue reservation.
